# The Analgesic Effect of the Mitochondria-Targeted Antioxidant SkQ1 in Pancreatic Inflammation

**DOI:** 10.1155/2016/4650489

**Published:** 2016-05-04

**Authors:** Maximilian Weniger, Leonard Reinelt, Jens Neumann, Lesca Holdt, Matthias Ilmer, Bernhard Renz, Werner Hartwig, Jens Werner, Alexandr V. Bazhin, Jan G. D'Haese

**Affiliations:** ^1^Department of General, Visceral, Transplantation, Vascular and Thoracic Surgery, Ludwig Maximilians University, Campus Grosshadern, 81377 Munich, Germany; ^2^Institute of Pathology, Ludwig Maximilians University, 81377 Munich, Germany; ^3^Institute of Laboratory Medicine, Ludwig Maximilians University, 81377 Munich, Germany

## Abstract

*Background*. Chronic pancreatitis is one of the main risk factors for pancreatic cancer. In acute and chronic pancreatitis, oxidative stress is thought to play a key role. In this respect, the recently described mitochondria-targeted antioxidant SkQ1 effectively scavenges reactive oxygen species at nanomolar concentrations. Therefore, we aimed to characterize the influence of SkQ1 on tissue injury and pain in acute and chronic pancreatitis.* Methods*. Both acute and chronic pancreatitis were induced in C57BL/6 mice by intraperitoneal cerulein injections and treatment with SkQ1 was carried out by peroral applications. Hyperalgesia was assessed by behavioral observation and measurement of abdominal mechanical sensitivity. Blood serum and pancreatic tissue were harvested for analysis of lipase and histology.* Results*. SkQ1 did not influence pain, serological, or histological parameters of tissue injury in acute pancreatitis. In chronic pancreatitis, a highly significant reduction of pain-related behavior (*p* < 0.0001) was evident, but histological grading revealed increased tissue injury in SkQ1-treated animals (*p* = 0.03).* Conclusion*. After SkQ1 treatment, tissue injury is not ameliorated in acute pancreatitis and increased in chronic pancreatitis. However, we show an analgesic effect in chronic pancreatitis. Further studies will need to elucidate the risks and benefits of mitochondria-targeted antioxidants as an analgesic.

## 1. Introduction

Acute and chronic pancreatitis rank within the most common causes for hospital admission among gastrointestinal disorders and represent a significant healthcare burden [1]. Furthermore, chronic pancreatitis represents a key risk factor predisposing to pancreatic cancer [2]. Despite intensive efforts, treatment is limited to supportive measures and none of the therapeutic approaches evaluated up until now have been shown to ameliorate the course of both acute and chronic pancreatitis. Oxidative stress has been proposed as a key pathophysiological factor [3, 4]. A common model of oxidative stress in pancreatitis suggests that oxidative stress is the result of cytochrome P450 induction, excess exposure to bioactivated metabolites, and a deficiency of reduced glutathione [3]. While evidence from animal studies suggests that antioxidant therapy could reduce inflammatory processes in pancreatitis [4–6], clinical studies were not able to demonstrate a relevant therapeutic effect [7, 8].

In chronic pancreatitis, especially, pain is a major clinical problem that affects up to 90% of patients and severely impacts quality of life [9, 10]. Furthermore, recurrent episodes of pancreatitis and pain are a relevant clinical problem in patients with pancreatic cancer [2]. Historically, pain in chronic pancreatitis has been thought to be caused by increased pancreatic pressure and mechanical strictures [11]. Current research on pain in chronic pancreatitis majorly focuses on pancreatic neuropathy and pancreatic neuritis [12]. In accordance with the hypothesis that oxidative stress is critically linked to the pathogenesis of chronic pancreatitis and its symptoms, numerous studies have attempted to demonstrate an analgesic effect of antioxidants in chronic pancreatitis [13]. In this respect, a recent Cochrane analysis demonstrated that antioxidants only slightly reduce pain in chronic pancreatitis and their clinical value remains unclear [13].

However, the effect of antioxidants in pancreatitis has only been evidenced by the use of classical, non-mitochondria-targeted antioxidants. Mitochondria-targeted antioxidants have recently been shown to exert cytoprotective effects [14–20] in numerous studies and are thought to be effective at nanomolar concentrations [21]. Within the cytosol, mitochondria are the only anionic organelles and are specifically targeted by the cationic group of antioxidants of this family [22]. In this regard, two antioxidants, SkQ1 [23] and MitoQ [24], have been the focus of research in the field of mitochondria-targeted antioxidants. Of these, SkQ1 has been shown to be effective at lower concentrations [23, 25] and has been demonstrated to ameliorate trauma-induced neurological deficit [16], protect erythrocytes from oxidative hemolysis [17], reduce TNF-*α* induced endothelial damage [18], modulate angiogenesis [26], and decrease ischemia-reperfusion injury in liver transplantation [20]. On a molecular level, the antioxidative effect of SkQ1 relies on plastoquinone, an electron carrier in photosynthesis [23].

As a highly effective scavenger of reactive oxygen species (ROS), we hypothesize that the mitochondria-targeted antioxidant SkQ1 [23] would reduce inflammatory cell damage in mouse models of acute and chronic pancreatitis. As such, serological and histological parameters were evaluated. Furthermore, we aimed to investigate whether SkQ1 would affect pancreatitis-associated pain as measured by behavioral pain testing.

## 2. Material and Methods

### 2.1. Animals

Female C57BL/6 mice obtained from Charles River (Sulzfeld, Germany) were kept in accordance with regulations of the Federal Republic of Germany with a 12-hour light-dark cycle and food and water ad libitum. All animal procedures were performed according to local ethical guidelines and approved by the district government of Upper Bavaria (55.2-1-54-2532-165-2014). Cerulein (50 *μ*g/kg/injection in saline; Sigma, Sigma Life Science, St. Louis, USA) or saline (control) was administered intraperitoneally 8 times at hourly intervals for the induction of acute pancreatitis. For experiments on chronic pancreatitis, mice received cerulein (50 *μ*g/kg/injection in saline) or saline (control) at five hourly injections of cerulein three times a week over a period of eight weeks. Antioxidative treatment with SkQ1 (10-(6′-plastoquinonyl)decyltriphenylphosphonium) [27] was administered perorally with the drinking water at a dose of 5 nmol/kg body weight per day (on average, a mouse drank about 5 mL of water per day).

For experiments on both acute and chronic pancreatitis, mice were divided in three groups. Group A (acute pancreatitis (AP) *n* = 8; chronic pancreatitis (CP) *n* = 12) was treated with 5 nmol/kg SkQ1, group B (AP *n* = 8; CP *n* = 12) was the untreated control, and group C (AP *n* = 8; CP *n* = 7) was the sham group, which was injected intraperitoneally with 0.9% NaCl instead of cerulein and was therefore the negative control group without pancreatitis.

For experiments on acute pancreatitis, mice were pretreated with SkQ1 for 8 weeks prior to induction of pancreatitis. Mice designated for experiments on chronic pancreatitis received SkQ1 at the same concentration for 8 weeks in parallel with induction of pancreatitis.

### 2.2. Open-Field Test/Vertical Activity

Behavioral measurement of nonevoked pain-related behavior was conducted as described before [28]. Originally, the open-field test was developed to measure emotionality by qualitative and quantitative measures of locomotion. Here, we focus on the vertical activity (rearing) as the main outcome parameter. Rearing is an explorative behavior that has been shown to mirror anxiety which closely correlates with pain sensation in rodents. Less vertical activity is, therefore, interpreted as more anxiety and pain. Briefly, mice were allowed to move freely in a square box of 70 × 70 × 60 cm. After an accommodation period of 10 minutes, behavior was recorded via a video camera over a time period of five minutes and vertical activity was determined by counting the times the mice reared up. Between the observations, the box was thoroughly cleaned with ethanol to eliminate olfactory cues from previous subjects. Vertical activity was assessed once before the first administration of cerulein and once after administration of cerulein was completed.

### 2.3. Von Frey's Test

Mechanical sensitivity and evoked pain-related behavior was measured using Von Frey's hairs. The mice were allowed to move freely in a transparent box of 10 × 10 × 10 cm placed on a metal-lattice. The boxes were cleaned with ethanol to eliminate odor from other subjects. Following an accommodation period of 10 minutes, the abdomen was stimulated with a series of Von Frey's hairs of 0.25, 0.5, 1.0, and 2.0 mN with 10 applications each. Reactions to stimulation with Von Frey's hairs were graded and a pain score was build. A response was defined as an arousal reaction and allocated with one point. Any type of defense or flight reaction was allocated with two points. A cumulative score representing the sum of pain scores of all filaments was used to assess mechanical hypersensitivity.

### 2.4. Serum Lipase

Blood samples were obtained via ventricular heart puncture before sacrifice. Serum lipase levels were measured in the central clinical laboratories (Zentrallabor, Hospital of the University of Munich, Germany) according to locally defined guidelines.

### 2.5. Histology

Hematoxylin and eosin staining of paraffin sections was performed according to standard protocols. Subsequently, slides were analyzed by an experienced pathologist (JN) and assessed according to Spormann's score [29, 30]. Tissue edema and neutrophil infiltration were both graded on a scale from zero to three, and parenchymal necrosis and fat necrosis were separately graded on a scale from zero to seven. The total score consisted of the sum of scores for tissue edema, neutrophil infiltration, parenchymal necrosis, and fat necrosis. Additionally, paraffin sections were stained with aniline blue to assess the degree of fibrosis.

### 2.6. Statistical Analysis

Statistical analysis was done using one-way ANOVA and Tukey's test for multiple comparisons. The *α*-level was set at 0.05. Results are displayed as means and their respective standard error of the mean (SEM). Data analysis was carried out using GraphPad Prism version 6.00 for Windows (GraphPad Software, La Jolla, California, USA, http://www.graphpad.com/).

## 3. Results

### 3.1. Acute Pancreatitis

#### 3.1.1. Nonevoked Pain-Related Behavior

**Figure 1:**
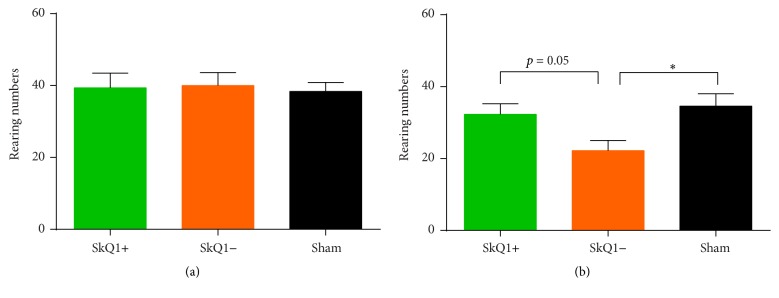
Vertical activity in acute (a) and chronic (b) pancreatitis: displayed are the rearing numbers that were counted during the observation time. While there were no differences between the groups in acute pancreatitis (a), treated mice (SkQ1+) with chronic pancreatitis showed significantly more activity than the untreated (SkQ1−) and saline controls (sham), suggesting less pain in the treated mice (b). ^*∗*^Statistically significant results (*p* < 0.05).

The number of observed rear ups was not statistically significant between both groups A (39.38 ± 4.09) and B (40.00 ± 3.65) or the sham group. Additionally, no statistically significant differences were evident between groups A and B (*p* = 0.99).  Figure 1(a) compares the vertical activity of all groups after induction of acute pancreatitis.

#### 3.1.2. Evoked Pain-Related Behavior

**Figure 2:**
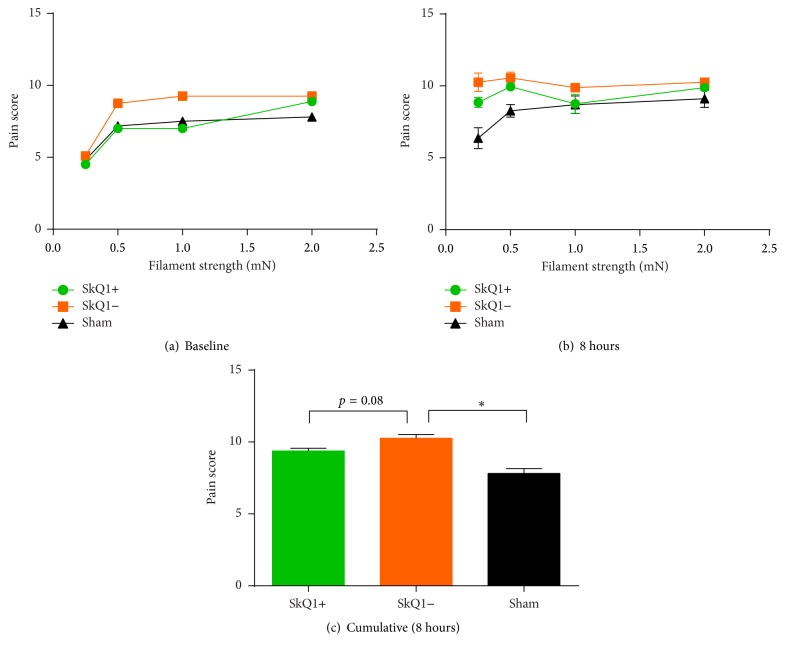
Evoked pain-related behavior measured by Von Frey's hairs in acute pancreatitis: both baseline and final measurements did not show any significant differences between the groups. ^*∗*^Statistically significant results (*p* < 0.05).

Baseline experiments did not reveal statistically significant differences between groups A, B, and C (Figure 2(a)). The cumulative pain scores of groups A and B were significantly different from the sham group (Figures 2(b) and 2(c)). However, we detected a tendency towards SkQ1 attenuating evoked pain in mice affected by pancreatitis. Pain scores for group A were 9.38 ± 0.20 and for group B 10.26 ± 0.27 (*p* = 0.08).

#### 3.1.3. Serum Lipase

**Figure 3:**
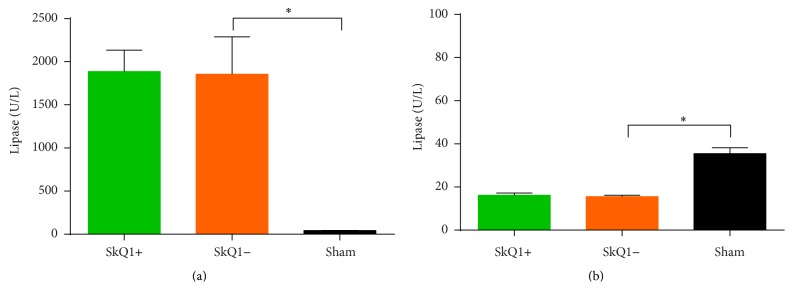
Serum lipase levels in acute (a) and chronic pancreatitis (b): while mice with acute pancreatitis showed significantly higher levels of serum lipase than the saline controls, no differences were observed with or without treatment (a). In chronic pancreatitis, mice showed lower serum lipase levels than the saline controls, reflecting advanced disease. As in acute pancreatitis, no differences were observed in treated versus untreated mice (b). ^*∗*^Statistically significant results (*p* < 0.05).

Serum lipase levels of groups A (1887 ± 247 U/L) and B (1856 ± 433 U/L) significantly differed from the sham group (Figure 3(a)). No statistically significant differences were seen in the comparison of groups A and B (*p* = 0.99). Figure 3(a) provides a graphical display of serum lipase levels in acute pancreatitis.

#### 3.1.4. Histological Grading

**Figure 4:**
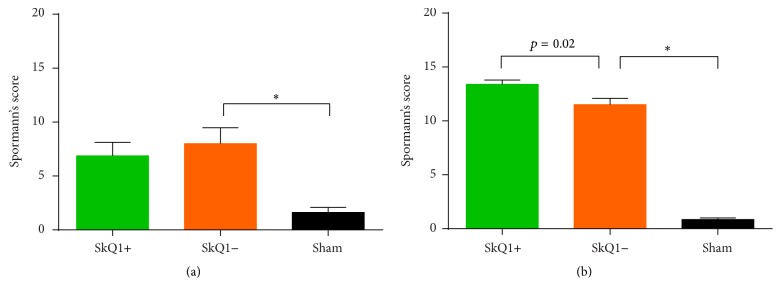
Histological severity of acute (a) and chronic pancreatitis (b) measured by Spormann's score: in acute pancreatitis, no differences were observed in treated versus untreated mice (a). In chronic pancreatitis mice treated with SkQ1 showed a slightly higher tissue damage when compared to the untreated mice (SkQ1−) (b). ^*∗*^Statistically significant results (*p* < 0.05).

**Figure 5 fig5:**
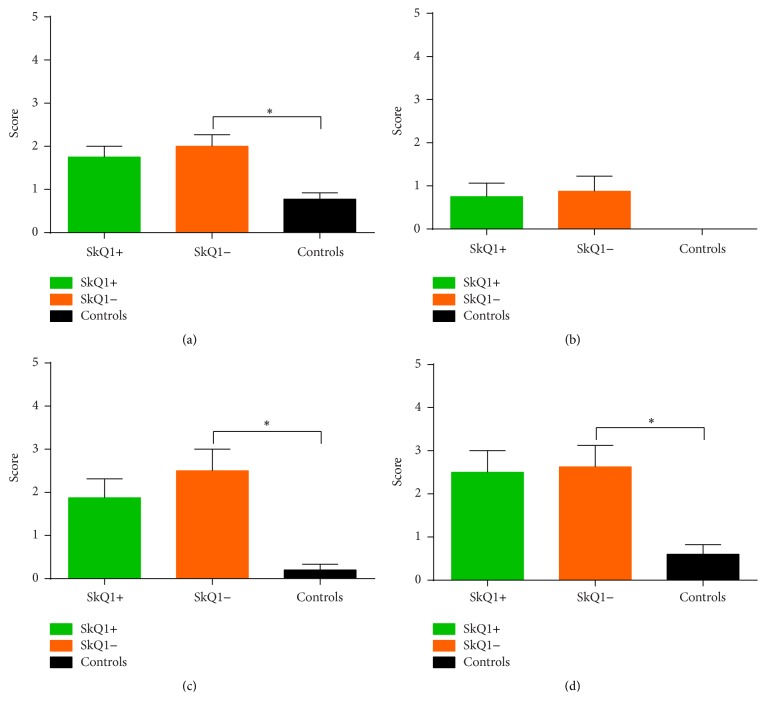
Subparameters of histological severity in acute pancreatitis as measured by Spormann's score: neither edema (a) nor neutrophil infiltration (b) nor parenchymal necrosis (c) nor fat necrosis (d) showed statistically significant differences between the SkQ1+ and SkQ1− groups. ^*∗*^Statistically significant results (*p* < 0.05).

**Figure 6 fig6:**
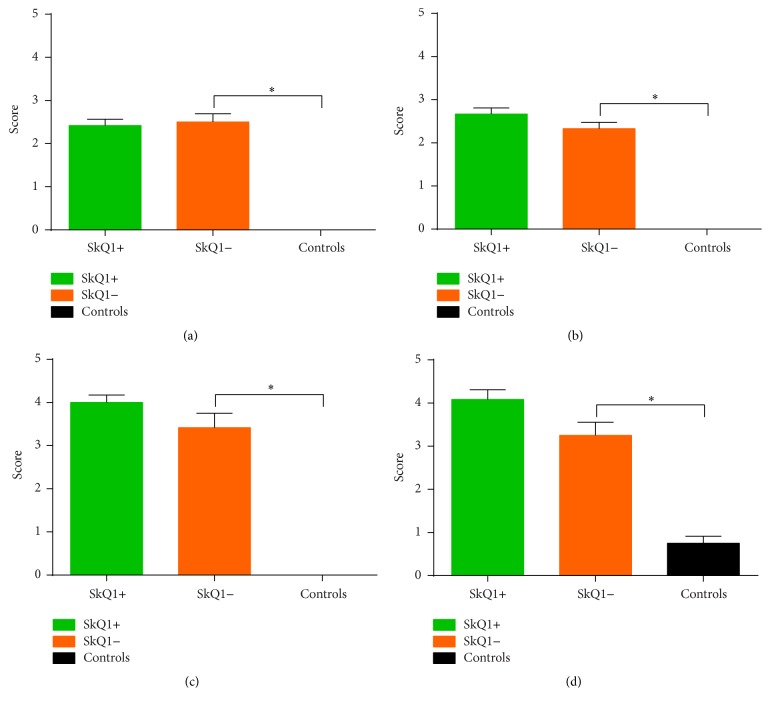
Subparameters of histological severity in chronic pancreatitis as measured by Spormann's score: again, none of the subparameters (edema (a), neutrophil infiltration (b), parenchymal necrosis (c), and fat necrosis (d)) showed statistically significant differences between the SkQ1+ and SkQ1− groups. ^*∗*^Statistically significant results (*p* < 0.05).

**Figure 7:**
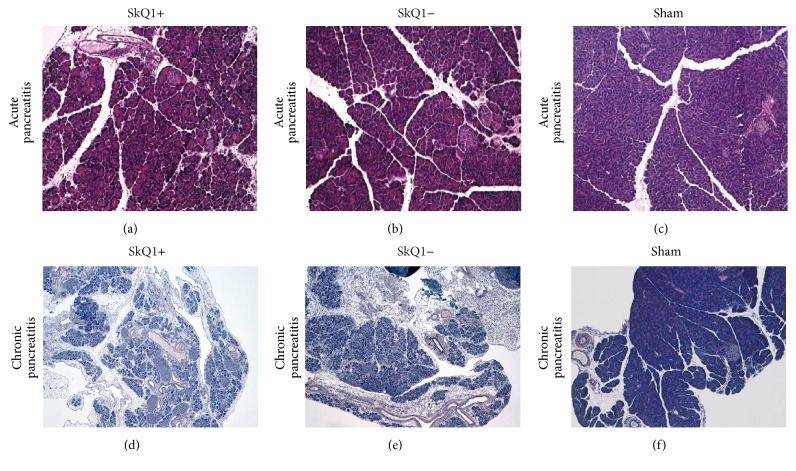
Representative HE staining of pancreatic tissue samples of mice with acute (a–c) and chronic pancreatitis (d–f).

As measured by Spormann's score (Figures 4(a) [Fig fig5] [Fig fig6] and 7(a)–7(c)), groups A and B displayed statistically significant differences from the sham group. The difference between groups A (6.87 ± 1.25) and B (8.00 ± 1.48) was not statistically significant (*p* = 0.77; Figures 4(a) [Fig fig5] [Fig fig6] and 7(a)–7(c)). Furthermore, a subanalysis of the parameters edema, neutrophil infiltration, parenchymal necrosis, and fat necrosis also did not show statistically significant differences between groups A and B (Figures 5(a)–5(d)).

### 3.2. Chronic Pancreatitis

#### 3.2.1. Nonevoked Pain

No statistically significant differences were evident at baseline measurements between groups A and B and the sham group. After week four, groups A and B showed statistically significant differences from the sham group, while a statistically significant difference between groups A (28.42 ± 2.82) and B (24.00 ± 3.38) was not seen (*p* = 0.62). Measurement of vertical activity at the end of the experiment at week eight (Figure 1(b)) revealed significantly increased vertical activity in group A with rearing of 32.25 ± 2.98 versus 22.17 ± 2.88 in group B (*p* = 0.05). While the mean of group B significantly differed from the sham group, the mean of group A did not (Figure 1(b)).

#### 3.2.2. Evoked Pain

**Figure 8:**
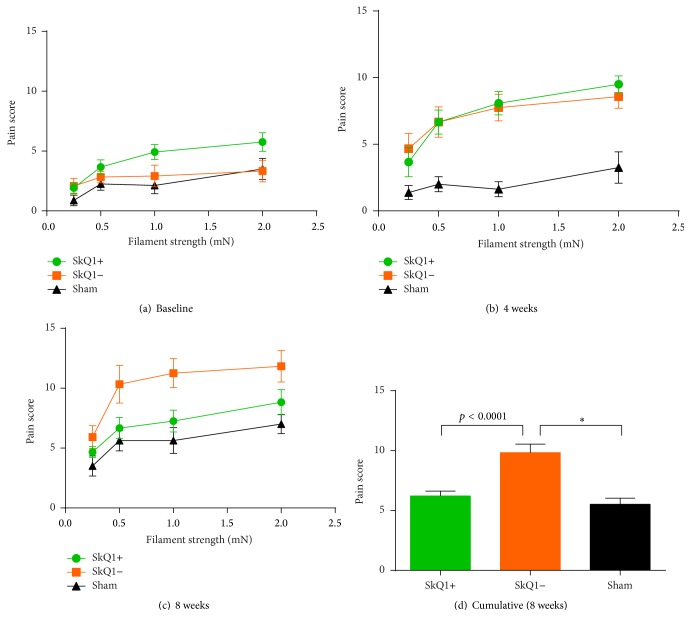
Evoked pain-related behavior measured by Von Frey's hairs in chronic pancreatitis: while baseline measurements did not differ significantly (a), mice with pancreatitis developed significantly increased pain scores after 4 weeks when compared to the saline controls (b). After 8 weeks of pancreatitis and 8 weeks of treatment, the SkQ1-treated mice show significantly less pain-related behavior than the untreated controls (c + d). ^*∗*^Statistically significant results (*p* < 0.05).

At baseline, no statistically significant differences were evident in the pain scores between both groups A and B and the sham group (Figure 8(a)). Moreover, mean pain scores between groups A (3.60 ± 0.35) and B (2.79 ± 0.37) also did not display statistically significant differences (*p* = 0.24; Figure 8(a)). After week four, still no difference between groups A (6.05 ± 0.50) and B (6.92 ± 0.55) was detected (*p* = 0.44; Figure 8(b)). But both groups A and B differed significantly from the sham group (Figure 8(b)). At week eight (Figures 8(c) and 8(d)), the cumulative pain score of group A (6.23 ± 0.39) significantly differed (*p* < 0.0001) from the pain score of group B (9.83 ± 0.70). When the sham group and group A were compared, no statistically significant difference was evident (*p* = 0.72; Figure 8(d)). The pain score of group B was statistically different from the sham group (*p* < 0.0001).

#### 3.2.3. Serum Lipase

Measurement of serum lipase did not reveal statistically significant differences between groups A (16.20 ± 1.00 U/L) and B (15.50 ± 0.74 U/L; *p* = 0.92; Figure 3(b)). Both groups A and B displayed statistically significant differences from the sham group. Figure 3(b) displays serum lipase levels of groups A–C.

#### 3.2.4. Histological Grading

Analysis of Spormann's score (Figures 4(b) [Fig fig5] [Fig fig6] and 7(d)–7(f)) revealed statistically significant differences between both groups A and B and the sham group. Spormann's score of group A was significantly higher than Spormann's score of group B (13.40 ± 0.40 versus 11.50 ± 0.58, resp.; *p* = 0.02; Figures 4(b) [Fig fig5] [Fig fig6] and 7(d)–7(f)). No statistically significant differences were detected with respect to the subparameters edema, neutrophil infiltration, parenchymal necrosis, and fat necrosis (Figures 6(a)–6(d)). Anilin staining for fibrosis confirmed the histological findings (data not shown).

## 4. Discussion

Chronic pancreatitis has been identified as a major risk factor for pancreatic cancer [2] and despite an abundance of studies and growing understanding of the pathophysiology of acute and chronic pancreatitis, there are still no specific therapeutic options [8, 31]. Experimental and clinical studies suggest a correlation of the oxidative burden and disease severity in acute pancreatitis [31, 32], which prompted numerous studies investigating the effect of antioxidants in acute and chronic pancreatitis [7]. One of the reasons for these disappointing previous results with antioxidants may be that they were only insufficiently able to penetrate mitochondria, one of the major sources for endogenous ROS production. To date, results are conflicting and, ultimately, do not point to a curative effect of antioxidants in pancreatitis [7, 8]. Nevertheless, mitochondria-targeted antioxidants have not been part of these investigations so far. Physiologically, ROS are byproducts of the mitochondrial oxygen metabolism [33] and are thought to interfere with apoptosis pathways [34]. In this respect, an imbalance of ROS and cellular antioxidative mechanisms has been linked to the pathogenesis and progression of acute and chronic pancreatitis. Mitochondria-targeted antioxidants have been shown to exert antioxidative effects at nanomolar concentrations inside mitochondria [23] and thus we hypothesized that scavenging ROS at their mitochondrial origin would reduce tissue injury in acute and chronic pancreatitis.

For acute pancreatitis, it has been shown that antioxidants may have a protective effect when administered as a pretreatment [5]. However, the results of the present study show that a pretreatment with SkQ1 does not result in reduced pain or tissue injury in cerulein-induced acute pancreatitis. The cerulein model for acute pancreatitis is the most frequently used model; however, it only induces a mild variant of acute pancreatitis over a very short time period of no more than 10 hours. It may, therefore, be possible that the influence of ROS and oxidative stress on pain and disease severity in acute pancreatitis is not as critical as initially hypothesized. SkQ1 exhibits a higher antioxidant activity than another structurally similar antioxidant—MitoQ [35]. Moreover, SkQ1 has in contrast to MitoQ a very wide therapeutic “window,” in the order of 10^3^, between its antioxidant and prooxidant effects [25]. Analogous to the results of the present study, Huang et al. demonstrated that the mitochondria-targeted antioxidant MitoQ does not improve tissue injury in a mouse model of acute pancreatitis [24]. Most interestingly, Huang et al. reported increased cell death rates in mice treated with MitoQ [24]. When comparing MitoQ to SkQ1, one has to consider that SkQ1 has a higher affinity to mitochondrial cardiolipin and has been demonstrated to quench ROS more effectively than MitoQ, and its antioxidant action was found to be exhibited even at nM concentrations [23, 25]. Taking these data and the results of Huang et al. [24] into account, a low, purely antioxidant concentration of 5 nmol/kg SkQ1 was considered sufficient in the present study. The same concentration has previously been shown to exert beneficial immunological effects in healthy [26] and pancreatic cancer bearing mice [26].

In chronic pancreatitis, pancreata of mice treated with SkQ1 display an even more severe disease when compared to untreated mice. In this respect, antioxidants have previously been described to hinder apoptosis and propel necrosis in pancreatitis [34]. Thus, a potential shift from apoptosis to necrosis might serve to explain increased tissue injury in experiments on chronic pancreatitis. Moreover, our results indicate that antioxidants do not only exert cytoprotective effects by scavenging ROS but rather interfere with a complex network of cellular messenger and effector proteins.

SkQ1 was not able to attenuate tissue injury in chronic pancreatitis, but mice treated with SKQ1 showed significantly less pain-related behavior. Among others, pain in chronic pancreatitis has been described as the result of peripheral neuropathy and neural damage [36, 37]. Furthermore, previous studies on mitochondria-targeted antioxidants clearly show neuroprotective effects of SkQ1 [16, 19]. We, therefore, hypothesize that SkQ1 treatment may be able to reduce the ROS-mediated intrapancreatic neural damage and thereby reduce pancreatic pain in chronic pancreatitis. In this respect, a recently published meta-analysis demonstrated a moderate pain reduction in chronic pancreatitis patients treated with antioxidants [38, 39]. Thus, an analgesic effect of SkQ1 in chronic pancreatitis may be postulated. However, this potential pain relief comes at the expense of increased tissue injury. The lack of parallelism between reduced pain and worsened tissue injury supports the hypothesis that the analgesic effect of SkQ1 is not mediated via inhibition of inflammatory processes within the pancreatic tissue. In this regard, amelioration of neural damage may be a possible mechanism. In pancreatic cancer, where pancreatic tissue injury is less important in advanced stages, mitochondria-targeted antioxidants might be an option as an auxiliary analgesic.

## 5. Conclusion

SkQ1 may aid in reducing pain in chronic pancreatitis. However, tissue injury in acute and chronic pancreatitis is not diminished by SkQ1. In the case of chronic pancreatitis, SkQ1 may increase disease severity. Thus, further studies are needed to identify the mechanism for increased tissue injury after SKQ1 in chronic pancreatitis and to elucidate the potential of SKQ1 as an analgesic in this setting.
